# Professional competences of the physiotherapists in the field of mental health in Portugal: a questionnaire based survey

**DOI:** 10.1080/07853890.2021.1896645

**Published:** 2021-09-28

**Authors:** Olena Brito, João Casaca Carreira

**Affiliations:** a Escola Superior de Saúde da Cruz Vermelha, Lisboa, Portugal; b Área de Ensino de Fisioterapia, Escola Superior de Saúde da Cruz Vermelha, Lisboa, Portugal

## Abstract

**Introduction:**

In 2001 the World Health Organisation made the following statement: "the Mental Health – neglected for far too long – is crucial to the overall well-being of individuals, societies and countries" [1]. In Portugal, the focus on this issue arose in 2017 when a national program for Mental Health was created stating that "people are living more years, but with disabilities on the area of Mental Health, which implies an overload for society" [2]. Currently, the importance of the role that Physiotherapy has in this field is demonstrated by the World Physiotherapy Day 2018 campaign held by World Confederation of Physical Therapy, which was centred on the theme "Physiotherapy and Mental Health" [3]. The role of the Physiotherapist in Mental Health aims to promote the welfare and autonomy of people with physical dysfunctions associated with mental diseases and use physical stimuli to influence Mental Health [4]. The purpose of this study is to characterise the professional profile of the Physiotherapists working in Portugal in the field of Mental Health.

**Materials and methods:**

This is questionnaire online-based survey. An online questionnaire was sent to institutions, hospitals, centres where official records showed physiotherapists working in the field of Mental Health. The questionnaire was divided in 2 parts: socio-demographic information and questions related to Mental Health practice and training. The questionnaire was answered by 18 physiotherapists, which had an average age of 38 years (±10.4) and 94% were female (*n* = 17). All the participants gave their informed consent.

**Results:**

All the respondents have at least 3 years of continuous work in Mental Health field and 50% (*n* = 9) of them have experience for more than 5 years. Physiotherapists intervene daily with a minimum of 3 patients and 56% (*n* = 10) of them work within a multi-professional team. Concerning education, 78% (*n* = 14) of the physiotherapists stated that the theme of Mental Health was insufficiently addresses during the bachelor degree. More than 70% (*n* = 12) of the physiotherapists declared that have none or a little support to their clinical practice concerning training, guidelines.

**Discussion and conclusions:**

Training in mental health during bachelor is insufficient and coincident with the information provided by the coordinators of the 19 physiotherapy degrees in Portugal. The recommendations collected in the questionnaire, highlight the main aspects that should be addressed within the mental health theme in the basic training of physiotherapists. Moreover, the physiotherapists working in the field report urgent needs of formation.
